# Genomic Epidemiology of Early SARS-CoV-2 Transmission Dynamics, Gujarat, India

**DOI:** 10.3201/eid2804.212053

**Published:** 2022-04

**Authors:** Jayna Raghwani, Louis du Plessis, John T. McCrone, Sarah C. Hill, Kris V. Parag, Julien Thézé, Dinesh Kumar, Apurva Puvar, Ramesh Pandit, Oliver G. Pybus, Guillaume Fournié, Madhvi Joshi, Chaitanya Joshi

**Affiliations:** University of Oxford, Oxford, UK (J. Raghwani, L. du Plessis, O.G. Pybus);; University of Edinburgh, Edinburgh, Scotland, UK (J.T. McCrone);; Royal Veterinary College, Hatfield, UK (S.C. Hill, O.G. Pybus, G. Fournié);; University of Bristol, Bristol, UK (K.V. Parag), Université Clermont-Auvergne, Saint-Genès-Champanelle, France (J. Thézé),; Gujarat Biotechnology Research Centre, Gandhinagar, India (D. Kumar, A. Puvar, R. Pandit, M. Joshi, C. Joshi)

**Keywords:** COVID-19, genomic epidemiology, severe acute respiratory syndrome coronavirus 2, SARS-CoV-2, coronaviruses, virus, coronavirus disease, virus transmission, transmission dynamics, phylogeography, respiratory infections, zoonoses, Gujarat, India

## Abstract

Limited genomic sampling in many high-incidence countries has impeded studies of severe respiratory syndrome coronavirus 2 (SARS-CoV-2) genomic epidemiology. Consequently, critical questions remain about the generation and global distribution of virus genetic diversity. We investigated SARS-CoV-2 transmission dynamics in Gujarat, India, during the state’s first epidemic wave to shed light on spread of the virus in one of the regions hardest hit by the pandemic. By integrating case data and 434 whole-genome sequences sampled across 20 districts, we reconstructed the epidemic dynamics and spatial spread of SARS-CoV-2 in Gujarat. Our findings indicate global and regional connectivity and population density were major drivers of the Gujarat outbreak. We detected >100 virus lineage introductions, most of which appear to be associated with international travel. Within Gujarat, virus dissemination occurred predominantly from densely populated regions to geographically proximate locations that had low population density, suggesting that urban centers contributed disproportionately to virus spread.

Global genomic surveillance of severe acute respiratory syndrome coronavirus 2 (SARS-CoV-2) has provided key insights into virus dissemination and evolution at local, national, and international scales. Detailed analysis of the epidemic in the United Kingdom during the first wave (*1*) showed major heterogeneity in the size and duration of different SARS-CoV-2 lineages, which was driven by high extinction rates and rapid fluctuations in virus importation. This pattern has been repeatedly observed globally; shifts in human mobility drastically alter the source of virus importations, and only a small proportion of importations lead to sustained community transmission (*2*–*6*). Virus importations can sometimes be sufficiently intense to have a major effect on epidemic dynamics (e.g., super-seeding of the Alpha variant in the United Kingdom) (*7*). Genomic surveillance has been fundamental in tracking these dynamics, including detecting the emergence and spread of novel variants that alter risk to public health (*8**,**9*).

Gujarat is the fifth largest state in India and the ninth most populated of its 28 states (>60 million inhabitants). Most of the population lives in rural areas (57.4%), although the proportion living in cities has been increasing in past decades because of urbanization (from 37.4% in 2001 to 42.6% in 2011) (*10*). Gujarat has an international border with Pakistan, as well as state borders with Rajasthan and Madhya Pradesh to the northeast and east and with Maharashtra and the Union Territories of Daman, Diu, and Nagar Haveli to the south. The region has 2 international airports, in Ahmedabad and Surat (Figure 1, panel A). The airport in Ahmedabad served ≈11 million passengers and the airport in Surat served ≈1.5 million passengers (*11*) during April 2019‒March 2020.

**Figure 1:**
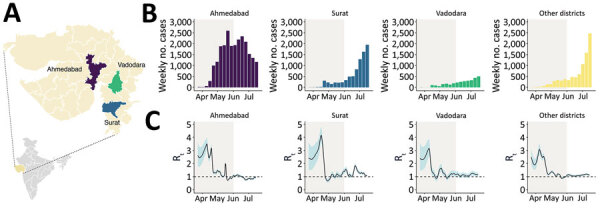
COVID-19 epidemiology during the first epidemic wave, Gujarat, India, 2020. A) Gujarat, highlighting the 3 most populous districts. Purple indicates Ahmedabad, blue indicates Surat, green indicates Vadodara, and yellow indicates all other districts. Inset map shows location of Gujarat in India. B) Weekly counts of newly reported cases of infection with COVID-19 for Ahmedabad, Surat, Vadodara, and other districts during April‒July 2020. C) Estimates of R_t_ for 4 locations on the basis of daily incidence data using 7-day averaging (see Methods). The black line indicates the median estimate, the blue shaded region indicates 95% equal-tailed Bayesian credible intervals, and the gray shaded region indicates the period of national lockdown. The dotted line indicates R_t_ = 1. COVID-19, coronavirus disease; R_t_, epidemic instantaneous reproduction number.

Although coronavirus disease (COVID-19) cases in India were first reported on January 30, 2020, and linked to travel from Wuhan, China, cases in Gujarat were initially identified on March 19, 2020 and were associated with travel from Saudi Arabia and the United Kingdom. A few days later, on March 24, 2020, a nationwide lockdown across India was announced. In Gujarat, special transportation (trains and buses) was arranged during March‒May 2020 to enable ≈1.8 million stranded migrant workers to return to their home states (i.e., outside Gujarat) or districts within Gujarat. Most specially organized trains originated from Gujarat (26%), which has one of the largest populations of migrant workers in India (*12*).

In this study, we investigated the introduction and transmission of SARS-CoV-2 in Gujarat during the first wave of the COVID-19 epidemic in India by using a combination of epidemiologic and genomic data. Through our analyses, we characterized the epidemiologic and lineage dynamics of SARS-CoV-2, evaluated key drivers of virus importation and transmission, and assessed whether major changes in human movement (i.e., because of lockdown and transport of migrant workers) shaped virus transmission dynamics in Gujarat. Our study demonstrates the limitations of current datasets and highlight the need for greater investment in virus genomic surveillance and collection of human mobility data to support comprehensive investigations into the origins and dissemination of future viral outbreaks.

## Methods

### Sample Collection, Library Preparation, Sequencing, and Data Analysis

During April‒July 2020, we generated 434 SARS-CoV-2 genome sequences for investigation. We collected nasopharyngeal/oropharyngeal swab specimens from COVID-19‒positive persons after obtaining informed consent and ethics approval. We transported samples and processed them for sequencing as described (*13*). In brief, we used the Ion AmpliSeq SARS-CoV-2 Research Panel and the Ion AmpliSeq Library Kit Plus (both from Thermo Fisher Scientific) for the library preparation. We performed sequencing on the Ion Torrent S5Plus system (Thermo Fisher Scientific) by using a 530 chip with 400-bp chemistry. We used a reference-based genome assembly, as described by Joshi et al. (*13*), to obtain whole-genome sequences. In brief, we used PRINSEQ-lite version 0.20.4 (*14*) for trimming and quality filtering. We mapped high-quality reads against a SARS-CoV-2 reference genome (GenBank accession no. NC_045512) by using CLC Genomics Workbench V 12.0 (QIAGEN, https://www.qiagen.com) to obtain consensus genomes.

### Epidemiologic Analysis

We obtained COVID-19 case data from a crowd-sourced initiative in India (https://www.covid19india.org and https://api.covid19india.org). These data were curated by volunteers from different data sources, such as state press bulletins and the Ministry of Health and Family Welfare of the Government of Gujarat. The source code is available in the GitHub repository (https://github.com/covid19india/api).

We estimated the instantaneous effective reproduction number at time t, R_t_ (median and 95% equal tailed Bayesian credible intervals), by using the EpiFilter method (*15*). This approach applies optimal recursive smoothing techniques to minimize the mean squared error in inferring R_t_ from the incidence of cases under a renewal transmission model. For all analyses, we assumed that the SARS-CoV-2 generation time distribution is well approximated by the serial interval distribution from (*16*), and we applied a weekly averaging filter to daily incidence data to reduce weekend effects and inconsistent reporting, which probably corrupts the incidence time series.

### Identifying Transmission Lineages

On the basis of lineages detected in the Gujarat SARS-CoV-2 dataset, we collated a representative global dataset comprising 10,000 genome sequences sampled evenly by week and country. To maximize our power to detect within-India dissemination, we added all sequences from India on GISAID (https://www.gisaid.org) as of November 5, 2020, to this dataset, for a final dataset size of 12,180 sequences (including samples from Gujarat). We followed a similar pipeline to that outlined in du Plessis et al. (*1*) and used ThorneyBeast (https://beast.community/thorney_beast) to estimate the posterior molecular clock tree. In brief, we estimated a maximum-likelihood tree by using iqtree (https://www.iqtree.org), and a Juke-Cantor substitution model with Wuhan/WH04/2020 as an outgroup. Branch lengths in this tree were scaled and rounded to represent the expected number of mutations. We then estimated the posterior tree distribution under a Skygrid coalescent prior (*17*) and a strict molecular clock model with a fixed rate (0.00075 substitutions/site/year). We executed 10 chains of 400 million steps, logging every 7.2 million steps, removing the first 40 million steps as burn-in.

We used a 3-state asymmetric discrete trait analysis (DTA) model implemented in BEAST version 1.10 (*18*). We used this analysis to infer the ancestral node locations (Gujarat, India, or global) by using an empirical tree distribution comprising 500 time-calibrated trees sampled from the posterior tree distributions estimated previously from ThorneyBeast.

To identify transmission lineages, we followed the method described by du Plessis et al. (*1*). In brief, starting from a randomly drawn Gujarat node in the maximum clade credibility (MCC) tree, we initiated a depth-first search, continuing until a non-Gujarat node was encountered or there were no additional nodes left to explore. The subtree visited during the search represents a transmission lineage or a singleton (if only 1 node was visited). We repeated this approach iteratively until all Gujarat nodes in the MCC tree had been visited. Using this approach, we identified 7 transmission lineages with >10 sequences, which we selected for further analysis.

### Drivers of Virus Transmission

To investigate the drivers of virus transmission, we focused on the 7 largest transmission lineages identified in the global dataset. Each dataset ranged from 11 to 75 sequences. We collated data on key predictors (population size and density, number of cases, geographic distance) at the district level. For each transmission lineage dataset, we used a separate exponential coalescent prior, while sharing a SRD06 substitutional model (*19*), a strict molecular clock model with a fixed rate (0.001 substitutions/site/year), and a generalized linear phylogeographic model (*20*). We executed 8 chains of 50 million steps (logged every 5,000 steps), then combined and thinned the chains by a factor of 10 after discarding the first 10% as burn-in.

## Results

Analysis of the instantaneous reproduction number over time (R_t_) of the epidemic indicated that the 3 most populous districts (Ahmedabad, Surat, and Vadodara) (Figure 1, panel A) showed rapid epidemic growth from late March through mid-April (Figure 1, panel C). However, after this initial period of growth, we observed a sharp decrease in R_t_ for all 3 major cities (Figure 1, panel C). The initial growth period in March and April occurred during the period of national lockdown. This finding might be a consequence of a time lag between the number of reported cases and the epidemiologic effect of the lockdown (which began on March 24, 2020) or might reflect considerable population movements during the early phase of the lockdown (e.g., resulting from persons returning to their home states or districts).

After the lockdown ended on June 1, 2020 (Figure 1), we observed slower epidemic growth in Ahmedabad, indicating that restriction in human mobility had a notable effect on disrupting chains of transmission. In contrast, in other districts, including Surat and Vadodara, from first week in June, the number of cases started to increase rapidly. Although Ahmedabad, Surat, and Vadodara recorded their first cases on March 19–20, 2020, Ahmedabad accounted for most confirmed cases during the first wave (Figure 1, panel B). This pattern is consistent with faster epidemic progression in Ahmedabad, possibly driven by higher frequency of virus importation compared with other districts, which have comparatively weaker international links because of lack of airports or greatly decreased passenger flow, and differences in population density and population connectedness.

**Figure 2:**
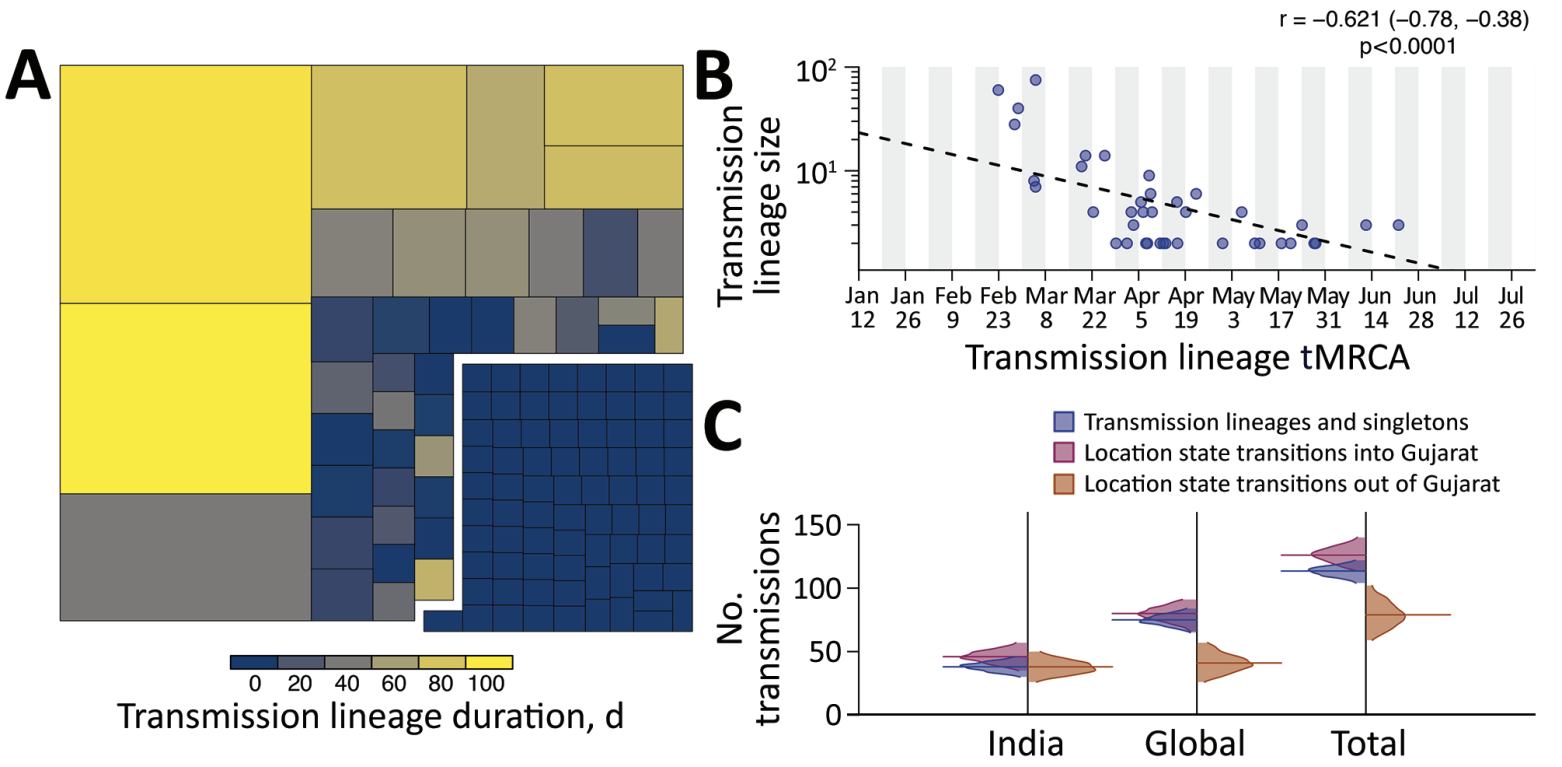
Size, duration, and importation of severe acute respiratory syndrome coronavirus 2 transmission lineages, Gujarat, India. A) Tree map summarizing the 113 detected transmission lineages by size. Colors indicate the duration of persistence of the lineage, and areas indicate the size of the transmission lineages. Lineage duration corresponds to time between the lineage’s oldest and most recently sampled genomes. B) Strong log‒linear relationship between size and mean tMRCA of each transmission lineages. Gray shading indicates time of testing; dashed line indicates slope. C) Breakdown of virus importations into Gujarat from other states in India or other countries. The number of location state transitions were estimated by using a robust counting approach (*21*) and a 3-location discrete trait phylogeographic analysis. tMRCA, time to most recent common ancestor.

To determine the source of virus importations in Gujarat, we characterized the lineage dynamics of SARS-CoV-2 in the region by using phylogenetic analyses. Specifically, we combined the 434 genome sequences generated from Gujarat with a representative subset of global genetic diversity of SARS-CoV-2 sampled over a similar period. We detected 39 (95% highest posterior density [HPD] 33–44) distinct Gujarat transmission lineages (defined as comprising >2 Gujarat genomes that belong to an ancestral lineage that originates from outside Gujarat), comprising 360 sequences, and 74 (95% HPD 63–83) singletons (genomes that could not be allocated to a Gujarat transmission lineage) across 2,000 posterior trees (Figure 2, panel A).

We identified 113 virus importations into Gujarat during the first wave. Consistent with previous analyses (*1*), larger transmission lineages were associated with longer duration times (the time between the oldest and most recently sampled genome within a transmission lineage), and smaller transmission lineages were associated with shorter duration times (Figure 2, panel A). This observation is further corroborated by the strong negative exponential relationship between transmission lineage size and the time to most recent common ancestor (tMRCA) (Figure 2, panel B). The mean tMRCA of transmission lineages was on April 17, 2020 (SD 35.8 days), and 75% of transmission lineages had a tMRCA from March 28 through May 7, 2020, which coincides with the period when the epidemic was increasing exponentially.

To determine likely sources of virus importation into Gujarat, we performed a 3-location discrete trait phylogeographic analysis (with location states of Gujarat, India [excluding Gujarat], and global [excluding India]). Despite having a border with other states with a high burden of disease (e.g., Maharashtra), our results suggest that virus importations into Gujarat had been driven primarily by international travel. However, the relatively low frequency of virus genome sampling across India is likely to mask importations from other states in India. Over the study period 3,092 SARS-CoV-2 genomes available from India were suitable for phylogenetic analysis (high-coverage and with complete temporal information), and only 930 were from neighboring states, predominantly from Maharashtra.

**Figure 3:**
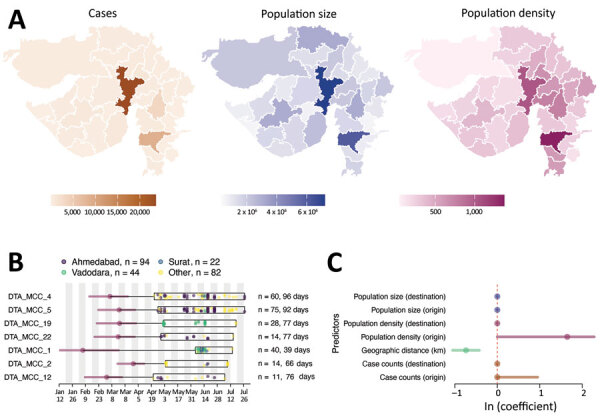
Determinants of SARS-CoV-2 lineage spread, Gujarat, India. A) Choropleth maps of key predictors (left, no. cases; middle, population size; right, population density, persons per square kilometer) that were evaluated in the phylogeographic generalized linear model analysis along with geographic distance. B) tMRCA and sample distribution of the 7 largest transmission lineages. For each lineage, circles correspond to the estimated lineage tMRCA, and horizontal bars indicate the 95% highest posterior density interval of the tMRCA. Box indicates date range of the samples from Gujarat for each lineage. Total number of samples and duration of each lineage are shown on the right. C) Predictors of SARS-CoV-2 lineage movement in Gujarat on the basis of 20 sampled districts. The contribution of each predictor is indicated by the mean coefficient value (points) and 95% highest posterior density interval (horizontal bars). tMRCA, time to most recent common ancestor. SARS-CoV-2, severe acute respiratory syndrome coronavirus 2.

Next, to evaluate the spatial dynamics of SARS-CoV-2 within Gujarat, we undertook a more detailed phylogeographic analysis to evaluate predictors of virus lineage dissemination by using a generalized linear model. To ensure the results reflect the specific dynamics of SARS-CoV-2 in Gujarat, we included only those sequences from the 7 largest Gujarat transmission lineages (Figure 3, panel B), which contained 11‒75 sequences. All 7 lineages were associated with virus importation from outside India and had a mean tMRCA that occurred close to the start of or before the national lockdown. Four lineages were composed mainly of genome sequences sampled from Ahmedabad, 2 were associated with Vadodara or Surat, and 1 was associated with the Aravalli District. The main predictors we tested were case counts, population size, and population density at both origin and destination locations and the road distance between districts (Figure 3, panel A).

Overall, our findings indicate that viral lineages moved more intensely between districts that were geographically closer (Bayes factor >100) (Figure 3, panel C) and predominantly from districts that had higher population density than districts that had lower population density (Bayes factor 40) (Figure 3, panel C). Together with the epidemiologic data (Figure 1, panel C), these results suggest that viral lineage movement was concentrated in exports from urban centers, which had higher caseloads and population densities, to nearby districts, initially from Ahmedabad but also at later stages from Surat and Vadodara. These movements contributed to the spread of SARS-CoV-2 within Gujarat.

**Figure 4:**
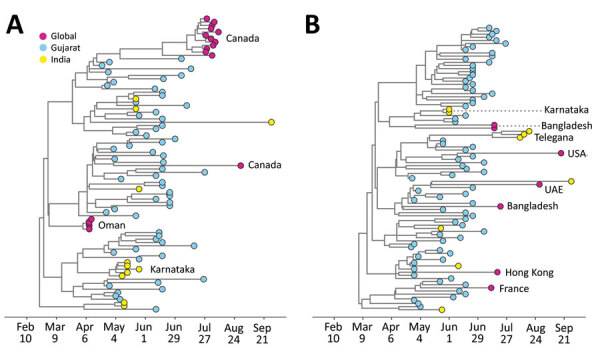
Timing of exportation of severe acute respiratory syndrome coronavirus 2 from Gujarat, India. Maximum clade credibility trees are for the 2 largest transmission lineages identified in this study: A) DTA_MCC_4; B) DTA_MCC_5. UAE, United Arab Emirates.

Although the global phylogeographic analysis suggested that importations of transmission lineages to Gujarat were associated mainly with international travel, phylogenetic analysis of the 2 largest Gujarat transmission lineages (Figure 4) provided evidence that these lineages were subsequently exported globally and to other states in India. Clusters (>2) of sequences from Canada, Oman, Karnataka, and Odisha were detected nested within DTA_MCC_4 (Figure 4, panel A), suggesting that this transmission lineage might have seeded outbreaks in these locations. Furthermore, in DTA_MCC_5, we detected clusters of sequences from Bangladesh and Telangana, as well as singletons from the United States, the United Arab Emirates, Hong Kong, and France. Several of these non-Gujarat clusters and singletons are on long branches, which indicates that we cannot exclude the possibility of intermediate locations in viral exportation. The mean tMRCAs of the 2 transmission lineages suggested probable importation before the first case in Gujarat was reported on March 19, 2020. However, given that the 95% HPD intervals overlapped with the start of the national lockdown (Figure 3, panel B) and low genomic sampling, these observations could reflect either multiple importations of closely related viruses from outside Gujarat (either another state in India or another country in which COVID-19 was circulating in early February or March) or a single importation into Gujarat before March 19, 2020, followed by cryptic community transmission. It is difficult to ascertain which scenario is more probable without additional data for human mobility and air travel, which is not available for the region.

## Discussion

Using epidemiologic data and virus whole-genome sequences, we investigated the spread, importation, and lineage dynamics of the first wave of SARS-CoV-2 in Gujarat, India. Initially, the epidemic was concentrated mainly in the most populous district, Ahmedabad, which accounted for more than half of reported cases. Epidemiologic analysis showed that R_t_ decreased across all districts after the national lockdown was announced. However, despite the slowdown in epidemic growth, the number of cases continued to increase across Gujarat after restrictions were gradually lifted on May 31, 2020, particularly in other populous districts, such as Surat and Vadodara. Phylogenetic analyses suggest that virus importations into Gujarat were comparatively higher than virus exportation from Gujarat and that virus importations were predominantly associated with international travel.

Virus lineage importations into Gujarat were associated mostly with global travel and visibly higher than either virus lineage importations from within India or viral exportations from Gujarat to other states in India, suggesting that mass transportation of migrant workers within and from Gujarat was not a major driver of virus transmission in Gujarat. However, the comparatively low genomic surveillance across India indicates that our study probably underestimated the number and rate of within-country virus movements. Nevertheless, our phylogeographic analysis identified geographic proximity between locations as a key driver of virus transmission in Gujarat, which was similarly observed for the Alpha variant in the United Kingdom (*7*). This finding suggests that disease control strategies should consider spatial context of SARS-CoV-2 spread (e.g., interventions should not only focus on populations with high disease prevalence but also be expanded to include geographically proximate populations to limit onward transmission). Particularly, more coordinated, responsive approaches at the local level could prevent traveling waves of infections without the need for national lockdowns (*22*). This strategy would probably require an improved understanding of multiscale mobility patterns across India and elsewhere to better respond to such epidemics (*23*).

As noted for the United Kingdom, virus importation is expected to occur earlier than the estimated tMRCA of a transmission lineage (0‒10 days) (*1*). However, because of a lack of detailed data on population movements into Gujarat, we were not able to estimate importation dates for the Gujarat transmission lineages. Nonetheless, the tMRCAs of transmission lineages serve as upper bounds on the importation events (i.e., importation must occur before the tMRCA). The earliest transmission lineages (based on tMRCA) were associated with importation from outside India, although because of the relatively small number of genomes from Gujarat, we could not evaluate changes in the dynamics of virus importation before and after the lockdown. However, similar to findings from previous studies (*1*), our results showed transmission lineages with earlier tMRCAs tended to be larger and have longer duration times.

Our analyses indicated that the Gujarat epidemic during the first wave was associated with >100 virus introductions. Given that this estimate was obtained from a relatively small number of sampled genomes, the true number is likely to be much larger. The faster progression of the epidemic in Ahmedabad compared with that in other districts strongly suggests this district was the epicenter of the first wave, probably enabled by a combination of higher inflow of international travelers, population density, and connectedness to the rest of Gujarat. Ahmedabad hosts the busiest airport in the state and is a major destination for interstate movement in Gujarat. Because incidence kept increasing after the gradual lifting of restrictions, suggesting that some transmission chains persisted during the lockdown and resumed growth once it ended, the focus of transmission started to shift to other districts, such as Surat and Vadodara, perhaps because of regional differences in human mobility and behavior (e.g., less movement or greater caution in Ahmedabad compared with other districts) (*12*). However, because of the limited temporal range of the sampled genomes, we could not test this hypothesis phylogenetically. Nevertheless, rapid advancement of epidemics in urbanized regions, followed by later movement into less populous regions, has been commonly observed elsewhere (*24**–**26*), including during the influenza A(H1N1) virus pandemic in 2009 (*27*). This finding strongly suggests that to reduce disease transmission, interventions should be implemented rapidly and robustly in major urban centers (e.g., as demonstrated in China during early 2020) (*28*).

Although our study period preceded the detection, emergence, and international spread of the Delta variant (Pango lineage B.1.617.2) in 2021, the findings about SARS-CoV-2 transmission dynamics from our study offer insight into how the Delta variant arose and spread within India and subsequently worldwide. We show the role of international connectedness and intraregional demographics in shaping virus lineage dynamics in India, and we highlight the limitations of investigating virus movement and origins because of comparatively low genomic surveillance in this region. All of these factors will need to be taken into consideration as part of evaluating the emergence and epidemiology of SARS-CoV-2 variants.

## References

[1] du Plessis L, McCrone JT, Zarebski AE, Hill V, Ruis C, Gutierrez B, et al.; COVID-19 Genomics UK (COG-UK) Consortium. Establishment and lineage dynamics of the SARS-CoV-2 epidemic in the UK. Science. 2021;371:708–12. 10.1126/science.abf2946 33419936 PMC7877493

[2] López MG, Chiner-Oms Á, García de Viedma D, Ruiz-Rodriguez P, Bracho MA, Cancino-Muñoz I, et al.; SeqCOVID-Spain consortium. The first wave of the COVID-19 epidemic in Spain was associated with early introductions and fast spread of a dominating genetic variant. Nat Genet. 2021;53:1405–14. 10.1038/s41588-021-00936-6 34594042 PMC8481935

[3] Murall CL, Fournier E, Galvez JH, N’Guessan A, Reiling SJ, Quirion P-O, et al. A small number of early introductions seeded widespread transmission of SARS-CoV-2 in Québec, Canada. Genome Med. 2021;13:169. 10.1186/s13073-021-00986-9 34706766 PMC8550813

[4] Lemieux JE, Siddle KJ, Shaw BM, Loreth C, Schaffner SF, Gladden-Young A, et al. Phylogenetic analysis of SARS-CoV-2 in Boston highlights the impact of superspreading events. Science. 2021;371:eabe3261. 10.1126/science.abe3261 33303686 PMC7857412

[5] Komissarov AB, Safina KR, Garushyants SK, Fadeev AV, Sergeeva MV, Ivanova AA, et al. Genomic epidemiology of the early stages of the SARS-CoV-2 outbreak in Russia. Nat Commun. 2021;12:649. 10.1038/s41467-020-20880-z 33510171 PMC7844267

[6] Tegally H, Wilkinson E, Lessells RJ, Giandhari J, Pillay S, Msomi N, et al. Sixteen novel lineages of SARS-CoV-2 in South Africa. Nat Med. 2021;27:440–6. 10.1038/s41591-021-01255-3 33531709

[7] Kraemer MUG, Hill V, Ruis C, Dellicour S, Bajaj S, McCrone JT, et al.; COVID-19 Genomics UK (COG-UK) Consortium. Spatiotemporal invasion dynamics of SARS-CoV-2 lineage B.1.1.7 emergence. Science. 2021;373:889–95. 10.1126/science.abj0113 34301854 PMC9269003

[8] Tegally H, Wilkinson E, Giovanetti M, Iranzadeh A, Fonseca V, Giandhari J, et al. Detection of a SARS-CoV-2 variant of concern in South Africa. Nature. 2021;592:438–43. 10.1038/s41586-021-03402-9 33690265

[9] Volz E, Mishra S, Chand M, Barrett JC, Johnson R, Geidelberg L, et al.; COVID-19 Genomics UK (COG-UK) consortium. Assessing transmissibility of SARS-CoV-2 lineage B.1.1.7 in England. Nature. 2021;593:266–9. 10.1038/s41586-021-03470-x 33767447

[10] Pandya RR, Raol MR, Mehta RA, Panchal JB, Chavda BK, Bhadarka KP, et al. Provisional population statistics as per 33 districts of Gujarat. Gujarat. Government of India; 2016 [cited 2022 Feb 24]. http://14.139.60.153/bitstream/123456789/13104/1/Statistical%20outline%20Gujarat%20state%202016.pdf

[11] Airport Authority of India. Traffic news for the month of March, 2020. p. 3 ‒5 [cited 2022 Feb 1]. https://www.aai.aero/en/business-opportunities/aai-traffice-news

[12] Ghosh RK, Tank N, Dutta M, Saxena S, Suthar P. Management of the COVID-19 pandemic in Gujarat: understanding the governance initiatives, leadership processes and their impact. Indian Institute of Management, Ahmedabad. 2020 [cited 2022 Feb 4]. https://www.iima.ac.in/c/document_library/Gujarat%20Covid%20Response%20Report-2020.pdf

[13] Joshi M, Puvar A, Kumar D, Ansari A, Pandya M, Raval J, et al. Genomic variations in SARS-CoV-2 genomes from Gujarat: underlying role of variants in disease epidemiology. Front Genet. 2021;12:586569. 10.3389/fgene.2021.586569 33815459 PMC8017293

[14] Schmieder R, Edwards R. Quality control and preprocessing of metagenomic datasets. Bioinformatics. 2011;27:863–4. 10.1093/bioinformatics/btr026 21278185 PMC3051327

[15] Parag KV. Improved estimation of time-varying reproduction numbers at low case incidence and between epidemic waves. PLOS Comput Biol. 2021;17:e1009347. 10.1371/journal.pcbi.1009347 34492011 PMC8448340

[16] Ferguson NM, Laydon D, Nedjati-Gilani G, Imai N, Ainslie K, Baguelin M, et al. Impact of non-pharmaceutical interventions (NPIs) to reduce COVID-19 mortality and healthcare demand. Imperial College London. 2020 [cited 2022 Feb 1]. https://www.imperial.ac.uk/mrc-global-infectious-disease-analysis/covid-19/report-9-impact-of-npis-on-covid-19/10.1007/s11538-020-00726-x PMC7140590 32270376

[17] Gill MS, Lemey P, Faria NR, Rambaut A, Shapiro B, Suchard MA. Improving Bayesian population dynamics inference: a coalescent-based model for multiple loci. Mol Biol Evol. 2013;30:713–24. 10.1093/molbev/mss265 23180580 PMC3563973

[18] Suchard MA, Lemey P, Baele G, Ayres DL, Drummond AJ, Rambaut A. Bayesian phylogenetic and phylodynamic data integration using BEAST 1.10. Virus Evol. 2018;4:vey016. 10.1093/ve/vey016 29942656 PMC6007674

[19] Shapiro B, Rambaut A, Drummond AJ. Choosing appropriate substitution models for the phylogenetic analysis of protein-coding sequences. Mol Biol Evol. 2006;23:7–9. 10.1093/molbev/msj021 16177232

[20] Lemey P, Rambaut A, Bedford T, Faria N, Bielejec F, Baele G, et al. Unifying viral genetics and human transportation data to predict the global transmission dynamics of human influenza H3N2. PLoS Pathog. 2014;10:e1003932. 10.1371/journal.ppat.1003932 24586153 PMC3930559

[21] Lemey P, Minin VN, Bielejec F, Kosakovsky Pond SL, Suchard MA. A counting renaissance: combining stochastic mapping and empirical Bayes to quickly detect amino acid sites under positive selection. Bioinformatics. 2012;28:3248–56. 10.1093/bioinformatics/bts580 23064000 PMC3579240

[22] Della Rossa F, Salzano D, Di Meglio A, De Lellis F, Coraggio M, Calabrese C, et al. A network model of Italy shows that intermittent regional strategies can alleviate the COVID-19 epidemic. Nat Commun. 2020;11:5106. 10.1038/s41467-020-18827-5 33037190 PMC7547104

[23] Balcan D, Colizza V, Gonçalves B, Hu H, Ramasco JJ, Vespignani A. Multiscale mobility networks and the spatial spreading of infectious diseases. Proc Natl Acad Sci U S A. 2009;106:21484–9. 10.1073/pnas.0906910106 20018697 PMC2793313

[24] Rader B, Scarpino SV, Nande A, Hill AL, Adlam B, Reiner RC, et al. Crowding and the shape of COVID-19 epidemics. Nat Med. 2020;26:1829–34. 10.1038/s41591-020-1104-0 33020651

[25] Viboud C, Bjørnstad ON, Smith DL, Simonsen L, Miller MA, Grenfell BT. Synchrony, waves, and spatial hierarchies in the spread of influenza. Science. 2006;312:447–51. 10.1126/science.1125237 16574822

[26] Dalziel BD, Kissler S, Gog JR, Viboud C, Bjørnstad ON, Metcalf CJE, et al. Urbanization and humidity shape the intensity of influenza epidemics in U.S. cities. Science. 2018;362:75–9. 10.1126/science.aat6030 30287659 PMC6510303

[27] Zachreson C, Fair KM, Cliff OM, Harding N, Piraveenan M, Prokopenko M. Urbanization affects peak timing, prevalence, and bimodality of influenza pandemics in Australia: Results of a census-calibrated model. Sci Adv. 2018;4:eaau5294. 10.1126/sciadv.aau5294 30547086 PMC6291314

[28] Lu J, du Plessis L, Liu Z, Hill V, Kang M, Lin H, et al. Genomic Epidemiology of SARS-CoV-2 in Guangdong Province, China. Cell. 2020;181:997–1003.e9. 10.1016/j.cell.2020.04.023 32359424 PMC7192124

